# Is the Multicolored Asian Ladybeetle, *Harmonia axyridis,* the Most Abundant Natural Enemy to Aphids in Agroecosystems?

**DOI:** 10.1673/031.013.15801

**Published:** 2013-12-24

**Authors:** Axel Vandereycken, Delphine Durieux, Emilie Joie, John J. Sloggett, Eric Haubruge, François J. Verheggen

**Affiliations:** 1 University of Liege, Gembloux Agro-Bio Tech, Entomologie fonctionnelle et évolutive, Passage des Déportés 2, B-5030 Gembloux, Belgium; 2 Kapoenstraat 2, 6211 KW Maastricht P.O. Box 616, 6200 MD Maastricht, The Netherlands

**Keywords:** aphidophagous insects, biological control, crop pests, Coccinellidae, invasive species

## Abstract

The multicolored Asian ladybeetle, *Harmonia axyridis* Pallas (Coleoptera: Coccinellidae), was introduced into Western Europe in the late 1990s. Since the late 2000s, this species has been commonly considered one of the most abundant aphid predators in most Western European countries. In spite of the large amount of research on *H. axyridis*, information concerning its relative abundance in agroecosystems is lacking. This study aims to evaluate the abundance of *H. axyridis* within the aphidophage community in four crops situated in southern Belgium: wheat, *Triticum aestivum* L. (Poales: Poaceae), corn, *Zea mays*, potato, *Solanum tuberosum* (Solanales: Solanaceae), and broad bean *Vicia faba* (Fabales: Fabaceae). In order to assess the species diversity, the collected data were analyzed by considering (1) the species richness and (2) the evenness according to the Shannon diversity index. Eleven aphidophages were observed in every inventoried agroecosystem, including five abundant species: three coccinellids, the seven-spotted ladybug, *Coccinella septempunctata* L. (Coleoptera: Coccinellidae), the 14-spotted Ladybird, *Propylea quatuordecimpunctata,* and *H. axyridis*; one hoverfly, the marmalade hoverfly, *Episyrphus balteatus* De Geer (Diptera: Syrphidae); and one lacewing, the common green lacewing, *Chrysoperla carnea* Stephens sensu lato (= s.l.) (Neuroptera: Chrysopidae). *Harmonia axyridis* has been observed to thrive, breed, and reproduce on the four studied crops. *Harmonia axyridis* is the most abundant predator of aphids in corn followed by *C. septempunctata,* which is the main aphid predator observed in the three other inventoried crops. In wheat and potato fields, *H. axyridis* occurs in low numbers compared to other aphidophage. These observations suggest that *H. axyridis* could be considered an invasive species of agrosystems, and that potato and wheat may intermittently act as refuges for other aphidophages vulnerable to intraguild predation by this invader. *Harmonia axyridis* is not the most abundant aphid predator in the main Belgian crops.

## Introduction

The multicolored Asian ladybeetle, *Harmonia axyridis* Pallas (Coleoptera: Coccinellidae), was introduced from Asia into Western Europe and other parts of the world to control aphid and coccid populations (Adriaens et al. 2008; Brown et al. 2008). In Belgium, *H. axyridis* was used as a biological control agent beginning in 1997 and was first observed in the wild in 2001. Since then *H. axyridis* populations have increased and gradually expanded into Belgium (Adriaens et al. 2008).

This species presents all the characteristics shared by an efficient aphid predator: large body size, high voracity, high predation efficiency (Labrie et al. 2006), high colonization aptitude (With et al. 2002), rapid development, high fecundity, and low susceptibility to pathogens or natural enemies (Marco et al. 2002). *Harmonia axyridis* has become ubiquitous in many parts of the world, including America, Europe, and Africa (Lombaert et al. 2010; Brown et al. 2011b), and has been reported in many different habitats, such as agroecosystems, gardens, and arboreal habitats (Majerus et al. 2006).

Due to its large body and efficient physical and chemical defenses, *H. axyridis* has become an intraguild predator (Sato and Dixon 2004; Ware and Majerus 2008). Intraguild predation has been observed among other ladybeetle species (Pell et al. 2008; Ware and Majerus 2008); other aphid natural enemies, including syrphids, chrysopids, and parasitoids (Phoofolo and Obrycki 1998; Wells et al. 2010; Ingels and De Clercq 2011); and aphid pathogenic fungus (Roy et al. 2008). This intraguild predation behavior is thought to have led to a decrease in native species (Brown and Miller 1998; Harmon et al. 2007; Ware et al. 2009; Brown et al. 2011a; Roy et al. 2012). In Belgian urban areas, Adriaens et al. (2010) found a significant decline in native species, including the two-spot ladybird, *Adalia bipunctata* L. (Coleoptera: Coccinellidae), and the 10 spotted ladybird, *Adalia decempunctata* L., on pine, lime, and maple trees following the arrival of *H. axyridis.* The decline of native species can possibly be explained by the decline in number of their principal prey, resulting in reduced survivorship in local habitats and altered dynamics of habitat use and dispersal (Evans 2004).

According to previous reports, the most dominant aphidophage in Belgian agroecosystems appear to be two coccinellids, the seven-spotted ladybug, *Coccinella septempunctata* L. (Coleoptera: Coccinellidae), and *H. axyridis*; one hoverfly, the marmalade hoverfly, *Episyrphus balteatus* De Geer (Diptera: Syrphidae); and one braconid, the parasitic wasp *Aphidius ervi* Haliday (Hymenoptera: Braconidae) (Derume et al. 2007; Adriaens et al. 2008; Alhmedi et al. 2009). In arboreal habitats, four coccinellids were reported as abundant species: *A. bipunctata*.,the 14-spotted ladybird, *Propylea quatuordecimpunctata* L. (Coleoptera: Coccinellidae), the 22-spot ladybird *Psyllobora vigintiduopunctata* (L.), and *H. axyridis* (Adriaens et al. 2008). In 2001, the same results were observed by Francis (2001), with the exception of *H. axyridis.*

Our study was conducted eight years after the first observation of *H. axyridis* in the wild in Belgium (Adriaens et al. 2003). Following aphidophagous decline highlighted by several studies, the current study was conducted in order to assess the relative abundance of *H. axyridis* through the quantification of the abundance of this exotic species and other aphidophages in four important Belgian crops (wheat, *Triticum aestivum* L. (Poales: Poaceae), corn, *Zea mays,* potato, *Solanum tuberosum* (Solanales: Solanaceae), and broad bean *Vicia faba* (Fabales: Fabaceae)) using a three-year inventory.

## Materials and Methods

### Study site

Aphidophagous insect populations were sampled from 2009 to 2011 in Hesbaye, an intensive agricultural production area in Wallonia, the southern region of Belgium (individual sites given in Table 1). Four crops were chosen for their agronomic importance: wheat, corn, potato, and broad bean *Vicia faba.* The sampling period ran from mid-May to late September. Every week, nine fields of each crop were sampled.

### Sampling methods

The sampling methods used to assess the numbers of aphidophagous predators and aphids consisted of whole-plant visual inspections, using 1 m^2^ quadrats distributed randomly throughout the whole field. In order to avoid the influence of surrounding crops, a 20 m buffer zone around the edge of each field was not sampled. Visual sampling was conducted, as it provides an easy and accurate method for the estimation of larval and adult densities of coccinellids in agroecosystems (Michels and Behle 1992). Forty-eight quadrats were examined in each crop every week. Quadrats were located along transect lines across each field and spaced 20 m apart. All leaves and stems within the quadrat were examined, and all aphidophagous stages were recorded. Aphid species were also quantified on all leaves and stems. Larvae and pupae were brought to the laboratory to develop under laboratory conditions (24 ± 1° C, 75 ± 5% RH) for identification at the species level. All aphid predators were identified, with the exception of members of the common green lacewing, *Chrysoperla carnea* Stephens (Neuroptera: Chrysopidae) species complex, which were pooled together. This group comprises three cryptic species, *C. kolthoffi* Navas, *C. lucasina* Lacroix, and *C. carnea* Stephens, which can only be differentiated using molecular techniques (Bozsik et al. 2003; Lourenco et al. 2006).

### Statistical analysis

In order to assess the species diversity, the collected data were analyzed by considering (1) species richness and (2) evenness according to the Shannon diversity index (H) (Magurran 1988), which considers both the number of species and the distribution of individuals among species. H is minimal if all individuals belong to only one species or if all species are represented by one individual; H is maximal if all individuals are evenly distributed. Evenness (J) varies from 0, if only one species dominates, to 1, if all species show similar abundance. Evenness (J) and the Shannon diversity index (H) were calculated as follows:


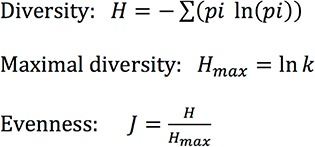


Because mean densities per m^2^ were low, these values are presented per 100 m^2^ The mean abundance per species was analyzed with an analysis of variance (ANOVA: general linear models) with crops (q = 4) and years (n = 3) used as factors (α = 0.05). Within crops, densities of species were compared using the least square difference (LSD; α = 0.05). To account for the variations of predator abundances, the abundance per species was analyzed using an analysis of covariance (ANCOVA: general linear models) with crops (q = 4) and years (n = 3) used as factors (α = 0.05) and aphid densities used as the covariable. Prior to analyses, a log_10_(x + 1) was used to transform the data distribution (counting) due to its asymmetry (Dagnelie 2011). Although statistical analyses were performed on transformed data, untransformed data are presented in Tables 4 and 5. Statistical analyses were performed using Minitab® release 1.5 (www.minitab.com).

## Results

### Diversity of aphidophages

During the three years (2009–2011), 11 aphidophagous taxa were observed on the four different cultures: the hoverfly *E. balteatus,* the coccinellids *C. septempunctata, C. quinquepunctata* L. (Coleoptera: Coccinellidae), *C. undecimpunctata* (L.), *H. axyridis, P. quatuordecimpunctata, A. bipunctata, A. decempunctata,* the cream-spot ladybird, *Calvia quatuordecimguttata* (L.), *Hippodamia variegata* Goeze; and the *C. carnea* species group (Table 2).

From 2009 to 2011, species richness increased in broad bean, corn, and wheat, reaching 6, 8, and 7 species respectively (Table 3). Species richness did not evolve in potato crops, remaining at five species during the entire period. Overall, five aphidophages were continually observed during the three-year period in each crop and represented 95% of all the aphidophage observed in 2009 and 99% in both 2010 and 2011: *E. balteatus, C. carnea* s.l., *C. septempunctata, P. quatuordecimpunctata,* and *H. axyridis.*

The evenness value was low in wheat with J = 0.35, 0.25, and 0.38 in 2009, 2010, and 2011, respectively, with the numerical dominance of two species: *C. septempunctata* and *E. balteatus* (Tables 3, 4, and 5). In broad bean and potato, evenness varied from year to year. In broad bean, *C. septempunctata* was the most abundant in 2010 (J = 0.45) and 2011 (J = 0.59). In potato, *C. carnea* s.l. and *C. septempunctata* numerically dominated the aphidophagous guild in all three years (J = 0.65, 0.79, 0.74) (Table 3). In corn, the evenness during the three years decreased from 0.82 (2009) to 0.61 (2010).

### Relative abundance of aphidophage in four crops

The relative abundance of both adult and larval stages of the five aphidophage within each crop showed significant differences (Tables 4 and 5), with the exception of potato in 2010, in which densities of larvae populations of different predators were not significantly different *(F_4.3570_* = 1.4*; p* = 0.25) (Table 5).

### Corn

The most abundant adult species in 2009 and 2010 was *C. carnea* s.l. and *C. septempunctata* (2009: *F_4,1065_* = 10.7, *p* < 0.001 ; 2010: *F_4,3425_* = 14.3, *p* < 0.001) *(p* < 0.05; LSD) (Table 4). In 2011, *H. axyridis* and *C. septempunctata* densities were both significantly higher than those of other species *(p* < 0.05; LSD) *(F_4,4055_* = 24.2, *p* < 0.001) (Table 4). *H. axyridis* larvae densities were significantly higher in 2009 and 2011 (2009: *F_4,1065_* = 5.4, *p* < 0.001 ; 2010: *F_4,3425_* = 25.5, *p* < 0.001 ; 2011: *F_4,4055_* = 57.0, *p* < 0.001) than those observed for the other species (Table 5).

### Wheat

Adult populations of *H. axyridis* remained lower than other species during the three-year sampling (2009: *F*_*4,1765*_ = 5.4, *p* < 0.001 ; 2010: *F_4,2925_* = 24.1*, p* < 0.001; 2011: *F_4,2625_* = 38.0, *p* < 0.001) *(p* < 0.05; LSD) and did not exceed 1.1 ± 0.6 adults per 100 m^2^ (Table 4). *Episyrphus balteatus* was the most frequently encountered adult species in 2009, whereas in 2010 and 2011 *C. septempunctata* was the most abundant species *(p* < 0.05; LSD) (Table 4). Larvae of *E. balteatus* were the most abundant *(p* < 0.05; LSD) of the aphidophage during the three years (2009: *F_4,1765_* =11.7, *p* < 0.001 ; 2010: *F_4,2925_ =* 91.1*, p* < 0.001; 2011: *F*_*4,2625*_ = 213.6, *p* < 0.001) (Table 5).

### Potato

Trends for *H. axyridis* were the same in potato as in wheat: *H. axyridis* was not the most abundant species, and its density did not exceed 2.5 ± 0.7 adults per 100 m^2^ (Table 4). Two species were more abundant than others: *C. carnea* s.l. in 2009 and 2011 (2009: *F*_*4,500*_ = 7.7, *p* < 0.001 ; 2011: *F_4,3250_* = 10.8, *p* < 0.001), and *C. septempunctata* in 2010 *(F_4,3570_* = 12.8, *p* < 0.001) and 2011 *(p* > 0.05; LSD). In 2009, larvae of *C. carnea* s.l., *P. quatuordecimpunctata,* and *H. axyridis* were the most abundant species but densities remained low (Table 5). In 2010, larvae densities of the five above-mentioned species were not significantly different from each other *(F_4,3570_* = 1.4, *p* = 0.25) (Table 5). In 2011, only *C. carnea* s.l. numerically dominated the aphidophages community (*F_4,3250_* = 5.5, *p* < 0.001) *(p* > 0.05; LSD) (Table 5).

### Broad bean

In 2009, *H. axyridis* and *C. septempunctata* adults were the only adult species observed in broad bean (*F*_*4,160*_ = 0.7, *p* > 0.05). *Coccinella septempunctata* was the most abundant species in 2010 *(F_4,2415_* = 22.1, *p* < 0.001), while in 2011 both *C. septempunctata* and *C. carnea* s.l. were profusely observed (*F_4,280_* = 37.5, *p* < 0.001) *(p* < 0.05; LSD) (Table 4). In 2009, three species were present at the larval stage: *P. quatuordecimpunctata, C. carnea* s.l., and *E. balteatus* (*F_4,160_* = 1.0 *,p* = 0.43) *(p* < 0.05; LSD). In 2010 (*F_4,2415_* = 8.35*, p* < 0.001) and 2011 *(F_4,2480_* = 21.2, *p* < 0.001), all species were observed, and *C. septempunctata* was the most abundant (Table 5).

### Effect of aphid densities and sampling year on relative abundance of aphidophage

Abundances of *H. axyridis* in wheat and potatoes were not analyzed, due to very low numbers of individuals observed during the three-year inventory. Over the three-year sampling, adult populations of *H. axyridis* in corn significantly increased (*F*_2,1709_ = 14.5, *p* < 0.001) (Table 6) from 7.8 ± 2.0 in 2009 to 19.9 ± 1.8 individuals per 100 m^2^ in 2011 (Table 4). Larval populations in the same crop also increased statistically (*F*_2,1709_ = 39.9, *p* < 0.001) (Table 6), rising from 11.9 ± 4.2 to 70.8 ± 6.6 larvae per 100 m^2^ (Table 5). In broad bean, relative abundance of *H. axyridis* was not significantly different among the three years, neither at the adult (Table 6) nor larval (Table 7) stages.

*Coccinella septempunctata* larvae declined in broad bean (F_2,1011_ = 4.7, *p* = 0.009), wheat (*F*_2,1463_ = 14.4, *p* < 0.001), and corn (*F*_2,1709_ = 31.9, *p* < 0.001) (Table 7); densities decreased by 10 and 29.6 times in corn and broad bean respectively. In wehat, no larvae were observed in 2011, while 13.4 ± 3.9 larvae per 100 m^2^ were observed in 2009.

The abundances of three other aphidophage showed variable changes (Tables 6 and 7).

### Aphid populations and correlation with aphidophage densities

In 2009, 2010, and 2011, seven, nine, and 10 species of aphids were identified, respectively: the pea aphid, *Acyrthosiphon pisum* Scopoli (Hemiptera: Aphididae); the cowpea aphid, *Aphis craccivora* Koch; the black bean aphid, *Aphis fabae* Scopoli; *Aphis frangulae* Walker; the buckthorn aphid, *Aphis nasturtii* Kaltenbach; the potato aphid, *Macrosiphon* *euphorbiae* Thomas; the vetch aphid, *Megoura viciae* Buckton; the rose grain aphid, *Metopolophium dirhodum* Walker; the green peach aphid, *Mizns persicae* Sulzer; *Rhopalosiphum* sp.; and *Sitobion* sp. (Table 8). The mean number of observed aphids increased in corn (*F_2,2589_* = 39., *p* < 0.001) and potato (*F_2,1410_* = 17.11, *p* < 0.001) from 2009 to 2011. Aphid densities also statistically varied in broad bean (*F_2,974_* = 8.7, *p* < 0.001) and wheat (*F_2,1392_* = 102.7, *p* < 0.001) from 2009 to 2011, but without any general evolution (Table 8).

The ANCOVA analyses showed a linear relationship between aphid and predator populations in 55% of adult populations (Table 6) and 35% of larvae populations (Table 7) (*p*_aphids_ < 0.05, ANCOVA). In these cases, aphid densities influenced the predator abundance. Results (*p*_years_) comparison between ANOVA and ANCOVA showed that the influence of aphid populations on predator abundance variations between years was not statistically significant.; *p_years_* of the two statistic analyses showed the same results.

## Discussion

Since the invasive coccinellid *H. axyridis* spread over Europe (Brown et al. 2008), imposing negative impacts on native aphidophage and affecting composition of several guilds (Soares et al. 2008; Roy et al. 2012), studies have evaluated the population spread of this coccinellid. The current study's sampling of aphid predators in Belgian agroecosystems from 2009 to 2011 showed that *H. axyridis* lives and reproduces more efficiently in corn and broad bean than in wheat and potato. In corn, the evenness during the three years decreased when *H. axyridis* population increased strongly and was higher than the population of other species.

During the three-year sampling, 11 aphidophage were observed in these agroecosystems, but five of them predominated: *E. balteatus, C. septempunctata, P. quatuordecimpunctata, H. axyridis,* and *C. carnea* s.l. Five dominant species in agroecosystems is a common observation (Hodek and Honěk 1996). Observations on predator densities highlight that *H. axyridis* was not the numerically dominant species in every crop: in wheat and potato, *C. septempunctata* was more abundant than *H. axyridis.* In many European agricultural crops, both *C. septempunctata* and *P. quatuordecimpunctata* were dominant prior to the arrival of *H. axyridis* (Honěk 1979; Bode 1980; Chambers et al. 1982), and it appears that these two species have maintained their dominance in spite of being prone to intraguild predation by *H. axyridis* in the field (Hautier et al. 2008).

In our study, larvae of *E. balteatus* were the most abundant observed predators in wheat, which has already been reported by Tenhumberg and Poehling (1995) prior to the arrival of *H. axyridis. Episyrphus balteatus* has also been previously reported as one of the most abundant aphidophage in vegetable crops, such as broad beans (Colignon et al. 2001; Colignon et al. 2002). This could be explained by abiotic conditions (high density cereal crop, with high humidity and low temperature) that are more favorable to the larvae of *E. balteatus* (Honěk 1983).

The fact that *H. axyridis* is not the most abundant aphidophage in agrosystems is probably due to its generalist behavior and arboreal habitat selection (Hodek 1973; Chapin and Brou 1991; LaMana and Miller 1996; Brown and Miller 1998; Labrie 2007). However, it has been reported that *H. axyridis* can also thrive in agrosystems such as wheat, corn, and potato (LaMana and Miller 1996; Buntin and Bouton 1997; Colunga-Garcia and Gage 1998; Michaud 2002; Brown 2003; Nault and Kennedy 2003; Snyder et al. 2004; Jansen and Hautier 2008), as well as in herbaceous habitats (LaMana and Miller 1996; Koch et al. 2006; Alhmedi et al. 2007).

There were evident changes in the abundance of aphidophages in crops through the years, but this study does not propose to identify a global evolution (increase or decline) in any of the species that were observed in this study. The causes of such fluctuations are diverse and may include factors such as the diversity and abundance of aphid species (Wright and Laing 1980; Honěk 1982; Thalji 2006). The results of the ANCOVA showed that there was a linear relationship between prey and predator populations, but additional biotic and abiotic factors contribute to the annual variability of predator abundance. Climate could be one such factor, due to its influence on natural enemies, overwintering mortality, and aphid populations (Hodek and Honěk 1996; Szentkirályi 2001; Rotheray and Gilbert 2011). Several other factors could also explain the variation between crops: insolation, humidity (Honěk 1985), quantity and quality of host plants (Alhmedi et al. 2009), and adjacent habitats (Colignon et al. 2001; Alhmedi et al. 2009).

A particularly interesting finding is that although *H. axyridis* breeding occurred in all four inventoried crops to some extent, adults of this species are not ubiquitous; few immature individuals were recovered from potato and wheat. Assuming that declines in native species are caused by *H. axyridis* (Roy et al. 2012), this suggests that certain crops, such as wheat and potato, could act as refuges from *H. axyridis* at certain times, while native species, such as *E. balteatus* and *C. septempunctata,* are able to breed with a lower risk of intraguild predation or other forms of competition from the invaders. Such habitats could become even more important as native species adapt to the invader by evolving to avoid habitats where *H. axyridis* occurs in high numbers, as has been seen in co-occurring aphidophages in their native habitats (Sloggett 2012).

In conclusion, our study indicates that *H. axyridis* was not the most frequently observed aphidophage in the four most important Belgian agronomical crops. In future studies, longer samplings would be preferable in order to eventually identify quantitative changes in the native fauna suggested from other studies. Agroecosystems may even constitute an ecological reservoir for certain native aphidophage.

**Table 1.**
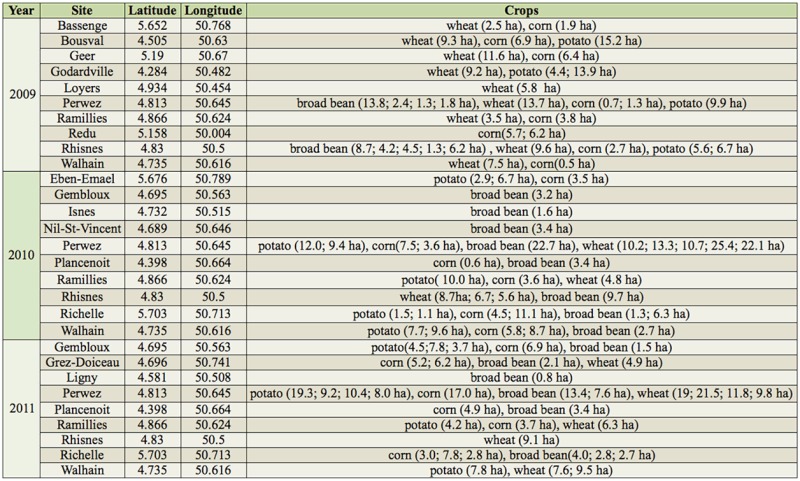
Sites surveyed for aphidophage from 2009 to 2011 in Belgium.

**Table 2.**
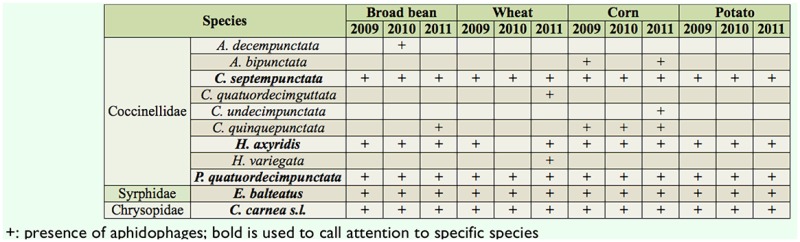
Aphidophage diversity in four crops (broad bean, wheat, corn, and potato) from 2009 to 2011.

**Table 3.**
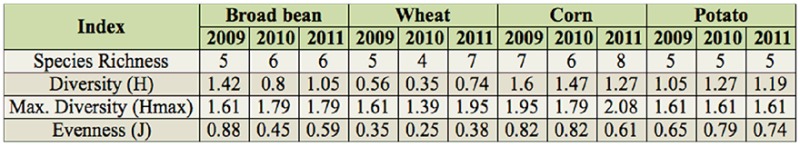
Species richness and diversity index (H= Shannon-Weiner diversity index, where absolute diversity = 1.00; J = evenness or relative diversity (H/Hmax), where absolute evenness = 1.00).

**Table 4.**
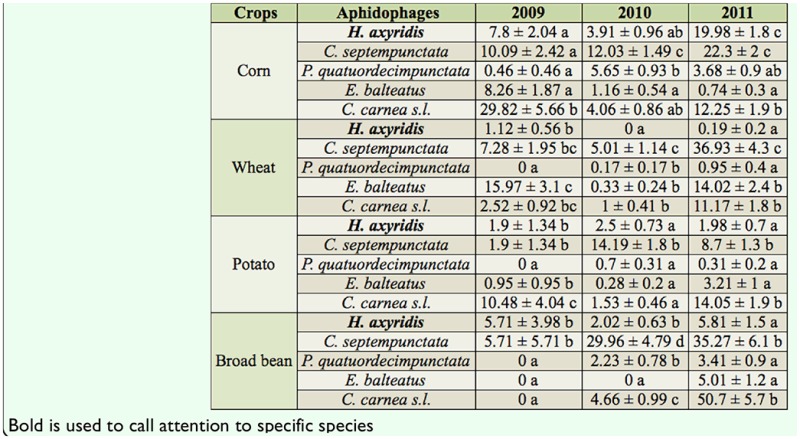
Abundance (means and SE) of aphidophage (adults) per 100 m^2^ in four agroeco system s from 2009 to 2011. Means within a crop followed by the same letter were not significantly different for the same field (*p* > 0.05; LSD test).

**Table 5.**
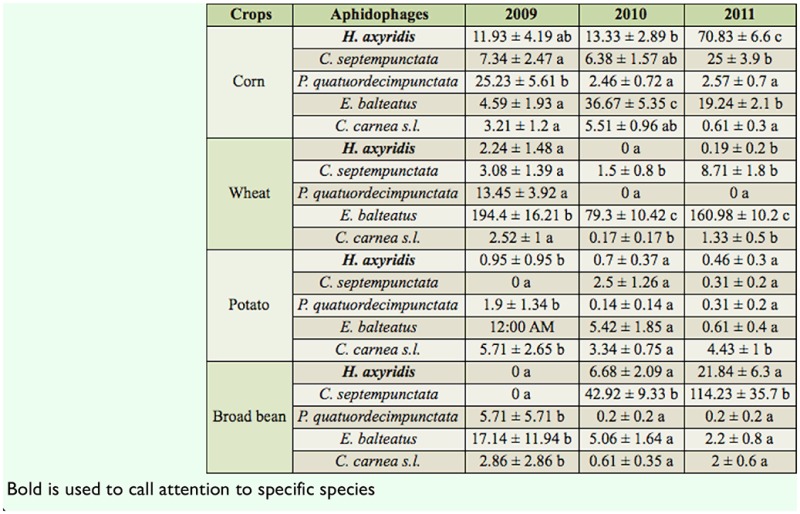
Abundance (means and SE) of aphidophage (larvae) per 100 m^2^ in four agroecosystems from 2009 to 2011. Means within a crop followed by the same letter were not significantly different for the same field (*p* > 0.05; LSD test).

**Table 6.**
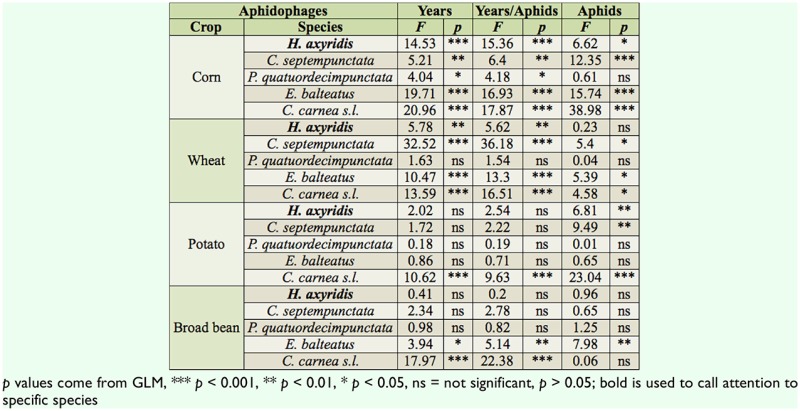
ANOVA and ANCOVA summary of the effects of aphid abundance and year sampling (2009, 2010, 2011) on the abundane of five predators at the adult stage in four crops (corn, wheat, potato, and broad bean).

**Table 7.**
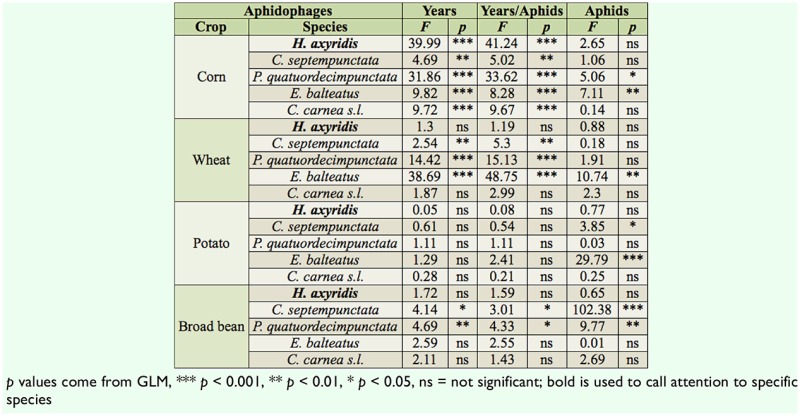
ANOVA and ANCOVA summary of the effects of aphid abundance and year sampling (2009, 2010, 2011) on the abundance of five predators at the larval stage in four crops (corn, wheat, potato, and broad bean).

**Table 8.**
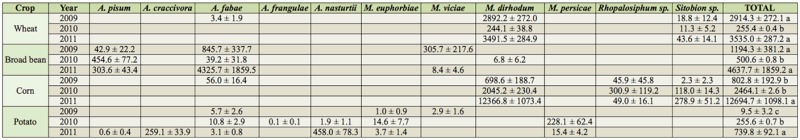
Mean numbers and SE of aphids/100 m^2^ observed in four fields (wheat, broad bean, corn, and potato) from 2009 to 2011.

## References

[bibr01] Adriaens T, Branquart E, Maes D. (2003). The Multicoloured Asian Ladybird *Harmonia axyridis* Pallas (Coleoptera: Coccinellidae), a threat for native aphid predators in Belgium?. *Belgian Journal of Zoology*.

[bibr02] Adriaens T, Gomez GMY, Maes D. (2008). Invasion history, habitat preferences and phenology of the invasive ladybird *Harmonia axyridis* in Belgium.. *BioControl*.

[bibr03] Adriaens T, San Martin G, Hautier L, Branquart E, Maes D. (2010). Toward a Noah's Ark for native ladybirds in Belgium.. *IOBC/wprs Bulletin*.

[bibr04] Alhmedi A, Haubruge E, Bodson B, Francis F. (2007). Aphidophagous guilds on nettle (*Urtica dioica*) strips close to fields of green pea, rape and wheat.. *Insect Science*.

[bibr05] Alhmedi A, Haubruge E, Francis F. (2009). Effect of stinging nettle habitats on aphidophagous predators and parasitoids in wheat and green pea fields with special attention to the invader *Harmonia axyridis* Pallas (Coleoptera: Coccinellidae).. *Entomological Science*.

[bibr06] Bode E. (1980). Aphids in winter wheat: abundance and limiting factors from 1976 to 1979.. *Bulletin SROP*.

[bibr07] Bozsik A, Mignon J, Gaspar C. (2003). The *Chrysoperla carnea* complex in Belgium (Neuroptera: Chrysopidae).. *Notes fauniques de Gembloux*.

[bibr08] Brown MW. (2003). Intraguild responses of aphid predators on apple to the invasion of an exotic species, *Harmonia axyridis*.. *BioControl*.

[bibr09] Brown MW, Miller SS. (1998). Coccinellidae (Coleoptera) in apple orchards of eastern West Virginia and the impact of invasion by *Harmonia axyridis*.. *Entomological News*.

[bibr10] Brown PMJ, Adriaens T, Bathon H, Cuppen J, Goldarazena A, Hagg T, Kenis M, Klausnitzer BEM, Kovar I, Loomans AJM, Majerus MEN, Nedved O, Pedersen J, Rabitsch W, Roy HE, Ternois V, Zakharov IA, Roy DB. (2008). *Harmonia axyridis* in Europe: spread and distribution of a non-native coccinellid.. *BioControl*.

[bibr11] Brown PMJ, Frost R, Doberski J, Sparks T, Harrington R, Roy HE. (2011a). Decline in native ladybirds in response to the arrival of *Harmonia axyridis*: early evidence from England.. *Ecological Entomology*.

[bibr12] Brown PMJ, Thomas CE, Lombaert E, Jeffries DL, Estoup A, Handley L-JL. (2011b). The global spread of *Harmonia axyridis* (Coleoptera: Coccinellidae): distribution, dispersal and routes of invasion.. *BioControl*.

[bibr13] Buntin GD, Bouton JH. (1997). Aphid (Homoptera: Aphididae) management in alfalfa by spring grazing with cattle.. *Journal of Entomological Science*.

[bibr14] Chambers RJ, Sunderland KD, Stacey DL, Wyatt IJ. (1982). A survey of cereal aphids and their natural enemies in winter-wheat in 1980.. *Annals of Applied Biology*.

[bibr15] Chapin JB, Brou VA. (1991). *Harmonia axyridis* (Pallas), the 3rd species of the genus to be found in the United-States (Coleopera, Coccineliidae).. *Proceedings of the Entomological Society of Washington*.

[bibr16] Colignon P, Gaspar C, Francis F. (2002). Effets de l'environnement proche sur la biodiversité entomologique en carottes de plein champ.. *Annales de la 2ème Conférence Internationale sur les moyens alternatifs de lutte contre les organismes nuisibles aux végétaux*.

[bibr17] Colignon P, Hastir P, Gaspar C, Francis F. (2001). Effets de l'environnement proche sur la biodiversité entomologique en cultures maraichères de plein champ.. *Parasitica*.

[bibr18] Colunga-Garcia M, Gage SH. (1998). Arrival, establishment, and habitat use of the multicolored Asian lady beetle (Coleoptera: Coccinellidae) in a Michigan landscape.. *Environmental Entomology*.

[bibr19] Dagnelie P. (2011). Les transformations de variables.. *Statistique théorique et appliquée. Tome 2. Inférence statistique à une et à deux dimensions.*.

[bibr20] Derume M, Hauteclair P, Bauffe C. (2007). Inventaire et comparaison de la faune des coccinelles (Coleoptera: Coccinellidae) des terrils des bassins miniers wallons liégeois et hennuyer (Belgique).. *Natura Mosana*.

[bibr21] Evans EW. (2004). Habitat displacement of North American ladybirds by an introduced species.. *Ecology*.

[bibr22] Francis F. (2001). Etude de la diversité et des plantes hôtes des Coccinellidae de Belgique.. *Notes fauniques de Gembloux*.

[bibr23] Harmon JP, Stephens E, Losey J. (2007). The decline of native coccinellids (Coleoptera: Coccinellidae) in the United States and Canada.. *Journal of Insect Conservation*.

[bibr24] Hautier L, Gregoire JC, de Schauwers J, Martin GS, Callier P, Jansen JP, de Biseau JC. (2008). Intraguild predation by *Harmonia axyridis* on coccinellids revealed by exogenous alkaloid sequestration.. *Chemoecology*.

[bibr25] Hodek I. (1973). *Biology of Coccinellidae.*.

[bibr26] Hodek I, Honěk A. (1996). *Ecology of Coccinellidae.*.

[bibr27] Honěk A. (1979). Plant -density and occurrence of *Coccinella septempunctata* and *Propylea quatuordecimpunctata* (Coleoptera: Coccinellidae) in cereals.. *Acta Entomologica Bohemoslovaca*.

[bibr28] Honěk A. (1982). Factors which determine the composition of field communities of adult aphidophagous coccinellidae (Coleoptera). *Journal of Applied Entomology*.

[bibr29] Honěk A. (1983). Factors affecting the distribution of larvae of aphid predators (Col., Coccinellidae and Dipt., Syrphidae) in cereal stands.. *Journal of Applied Entomology*.

[bibr30] Honěk A. (1985). Habitat preferences of aphidophagous coccinellids (Coleoptera).. *Entomophaga*.

[bibr31] Ingels B, De Clercq P. (2011). Effect of size, extraguild prey and habitat complexity on intraguild interactions: a case study with the invasive ladybird *Harmonia axyridis* and the hoverfly *Episyrphus balteatus*.. *BioControl*.

[bibr32] Jansen J, Hautier L. (2008). Ladybird population dynamics in potato: comparison of native species with an invasive species, *Harmonia axyridis*.. *Biological Control to Invasion*.

[bibr33] Koch RL, Venette RC, Hutchison WD. (2006). Invasions by *Harmonia axyridis* (Pallas) (Coleoptera: Coccinellidae) in the Western Hemisphere: implications for South America.. *Neotropical Entomology*.

[bibr34] Labrie G. (2007). Les mécanismes d'invasion de la coccinelle asiatique *Harmonia axyridis* Pallas au Québec.. *Biologie*.

[bibr35] Labrie G, Lucas E, Coderre D. (2006). Can developmental and behavioral characteristics of the multicolored Asian lady beetle *Harmonia axyridis* explain its invasive success?. *Biological Invasions*.

[bibr36] LaMana ML, Miller JC. (1996). Field observations on *Harmonia axyridis* Pallas (Coleoptera: Coccinellidae) in Oregon.. *Biological Control*.

[bibr37] Lombaert E, Guillemaud T, Cornuet JM, Malausa T, Facon B, Estoup A. (2010). Bridgehead Effect in the Worldwide Invasion of the Biocontrol Harlequin Ladybird.. *PLOS ONE*.

[bibr38] Lourenco P, Brito C, Backeljau T, Thierry D, Ventura MA. (2006). Molecular systematics of the *Chrysoperla carnea* group (Neuroptera: Chrysopidae) in Europe.. *Journal of Zoological Systematics and Evolutionary Research*.

[bibr39] Magurran AE. (1988). *Ecological diversity and its measurement.*.

[bibr40] Majerus M, Strawson V, Roy H. (2006). The potential impacts of the arrival of the harlequin ladybird, *Harmonia axyridis* (Pallas) (Coleoptera: Coccinellidae), in Britain.. *Ecological Entomology*.

[bibr41] Marco DE, Paez SA, Cannas SA. (2002). Species invasiveness in biological invasions: a modelling approach.. *Biological Invasions*.

[bibr42] Michaud JP. (2002). Invasion of the Florida citrus ecosystem by *Harmonia axyridis* (Coleoptera: Coccinellidae) and asymmetric competition with a native species, *Cycloneda sanguinea*.. *Environmental Entomology*.

[bibr43] Michels GJ, Behle RW. (1992). Evaluation of sampling methods for lady beetles (Coleoptera: Coccinellidae) in grain-sorghum.. *Journal of Economic Entomology*.

[bibr44] Nault BA, Kennedy GG. (2003). Establishment of multicolored Asian lady beetle in Eastern North Carolina: seasonal abundance and crop exploitation within an agricultural landscape.. *BioControl*.

[bibr45] Pell JK, Baverstock J, Roy HE, Ware RL, Majerus MEN. (2008). Intraguild predation involving *Harmonia axyridis*: a review of current knowledge and future perspectives.. *BioControl*.

[bibr46] Phoofolo MW, Obrycki JJ. (1998). Potential for intraguild predation and competition among predatory Coccinellidae and Chrysopidae.. *Entomologia Experimentalis Et Applicata*.

[bibr47] Rotheray G, Gilbert FS. (2011). *The Natural History of Hoverflies.*.

[bibr48] Roy HE, Adriaens T, Isaac NJB, Kenis M, Onkelinx T, Martin GS, Brown PMJ, Hautier L, Poland R, Roy DB, Comont R, Eschen R, Frost R, Zindel R, Van Vlaenderen J, Nedvěd O, Ravn HP, Grégoire J-C, de Biseau J-C, Maes D. (2012). Invasive alien predator causes rapid declines of native European ladybirds.. *Diversity and Distributions*.

[bibr49] Roy HE, Baverstock J, Ware RL, Clark SJ, Majerus MEN, Baverstock KE, Pell JK. (2008). Intraguild predation of the aphid pathogenic fungus *Pandora neoaphidis* by the invasive coccinellid *Harmonia axyridis*.. *Ecological Entomology*.

[bibr50] Sato S, Dixon AFG. (2004). Effect of intraguild predation on the survival and development of three species of aphidophagous ladybirds: consequences for invasive species.. *Agricultural and Forest Entomology*.

[bibr51] Sloggett JJ. (2012). *Harmonia axyridis* invasions: Deducing evolutionary causes and consequences.. *Entomological Science*.

[bibr52] Snyder WE, Clevenger GM, Eigenbrode SD. (2004). Intraguild predation and successful invasion by introduced ladybird beetles.. *Oecologia*.

[bibr53] Soares AO, Borges I, Borges PAV, Labrie G, Lucas E. (2008). *Harmonia axyridis*: What will stop the invader?. *BioControl*.

[bibr54] Szentkirályi F., McEwen PK, New TR, Whittington AE (2001). Ecology and habitat relationships.. *Lacewings in the**Crop Environment*.

[bibr55] Tenhumberg B, Poehling HM. (1995). Syrphids as natural enemies of cereal aphids in Germany: aspects of their biology and efficacy in different years and regions.. *Agriculture, Ecosystems and Environment*.

[bibr56] Thalji R. (2006). Composition of coccinellid communities in sugar beet fields in Vojvodina.. *Zbornik Matice Srpske za Prirodne Nauke*.

[bibr57] Ware R, Yguel B, Majerus M. (2009). Effects of competition, cannibalism and intra-guild predation on larval development of the European coccinellid *Adalia bipunctata* and the invasive species *Harmonia axyridis*.. *Ecological Entomology*.

[bibr58] Ware RL, Majerus MEN. (2008). Intraguild predation of immature stages of British and Japanese coccinellids by the invasive ladybird *Harmonia axyridis*.. *BioControl*.

[bibr59] Wells PM, Baverstock J, Majerus MEN, Jiggins FM, Roy H, Pell JK. (2010). Intraguild predation of non-coccinellid aphid natural enemies by *Harmonia axyridis:* prey range and factors influencing intraguild predation.. *IOBC/wprs Bulletin*.

[bibr60] With KA, Pavuk DM, Worchuck JL, Oates RK, Fisher JL. (2002). Threshold effects of landscape structure on biological control in agroecosystems.. *Ecological Applications*.

[bibr61] Wright EJ, Laing JE. (1980). Numerical response of coccinellids to aphids in corn in Southern Ontario.. *Canadian Entomologist*.

